# Personalized gene expression prediction in the era of deep learning: a review

**DOI:** 10.1093/bib/bbag022

**Published:** 2026-01-30

**Authors:** Viksar Dubey, Li Shen

**Affiliations:** College of Computing, Data Science, and Society, University of California, 2195 Hearst Avenue, Berkeley, CA 94720, United States; Department of Artificial Intelligence and Human Health, Icahn School of Medicine at Mount Sinai, 1 Gustave L. Levy Place, New York, NY 10029, United States; Department of Neuroscience, Icahn School of Medicine at Mount Sinai, 1 Gustave L. Levy Place, New York, NY 10029, United States

**Keywords:** gene expression, deep learning, personalized genomics, genetic variants, genome AI

## Abstract

Predicting gene expression from genomic sequences is a central goal in computational genomics. Recent advances have demonstrated that deep learning models trained on large-scale epigenomic datasets hold significant promise for this task. However, their success heavily depends on how they are applied: most models are trained exclusively on a reference genome, limiting their ability to capture individual-specific genetic variation. Consequently, while these models perform well on reference genomes, they often struggle when applied to personal genomic data. This review discusses recent efforts to overcome these limitations and explores methods aimed at improving the prediction of personalized gene expression. In particular, we compare the performance of deep learning models with traditional expression quantitative trait loci-based linear approaches, examining novel fine-tuning strategies, and highlighting the emergence of genomic language models. Across multiple studies, we find that deep learning models still face significant challenges in outperforming linear models for cross-individual gene expression prediction. Despite ongoing advances in model architecture and training methodology, accurately and robustly predicting personalized gene expression remains an open challenge in the field.

## Introduction

Predicting gene expression has become a central objective in computational genomics over the past decade [1]. Accurate prediction and analysis of gene expression enable researchers to better understand the role of regulatory variants in gene function and activation, as well as the broader relationship between genotype and phenotype [2]. By linking genetic variants to quantitative changes in gene expression, researchers can uncover how mutations contribute to disease mechanisms in relevant cell types and facilitate the development of personalized medical treatments tailored to an individual’s genetic profile.

With growing interest in gene expression prediction, machine learning (ML) has emerged as the leading framework for modeling gene regulatory activity. Traditional ML approaches—such as regularized linear regressions—have been widely used to “impute” gene expression from predefined genetic variants [3–7]. These models, however, are limited by their relatively simple architectures, which constrain their ability to generalize to unseen variants and to capture the complex, nonlinear cis-regulatory relationships. They are typically trained using expression quantitative trait loci (eQTLs)—including single nucleotide variants (SNVs) and small insertions or deletions (indels)—that are statistically associated with variation in gene expression.

In recent years, deep learning has emerged as a promising alternative to addressing the limitations of classical ML models [8]. Deep learning models can be trained directly on raw genomic sequences and can make predictions from arbitrary input sequences, allowing them to generalize to previously unseen variants. Their ability to capture complex regulatory relationships is supported by their success in other fields, such as computer vision [9] and natural language processing [10]. While deep learning models excel at predicting gene expression across genes within an individual, recent evaluations have shown that linear models often outperform deep learning approaches in predicting gene expression across individuals [11, 12]. This counterintuitive result may stem from the robustness of linear models in capturing the genetic basis of expression variation under noisy data conditions, despite their simpler structures.

The goal of this review is to provide an overview of ML approaches developed for personal-level sequence-to-expression prediction, with a particular focus on recent advances in deep learning. We will highlight strategies that have been employed to address the current limitations of deep learning models and explore the emergence of genomic language models and their potential applications to this problem.

## Classic machine learning methods

Classic ML methods, such as linear models [3–7] and random forest [13], have long been used to predict gene expression in the field of eQTL. These models are trained on SNVs and small indels, rather than raw genomic sequences, for the prediction of gene expression. The genetic variants that are associated with expression are known as eQTLs. One of the first, and still widely used, linear models is PrediXcan [3]. Although nonlinear models such as random forest [13] have been used for eQTL analysis, we find linear models to be much more dominant in the field due to their robust performance and easy interpretation. Therefore, this review will focus on linear models and their effectiveness compared to deep learning models.

To use classic ML methods, the features need to be constructed from predefined genetic variants; thereby, the input to a model is $X=[X_{1},X_{2},...X_{m}]$ where $X_{i}$ represents a SNV or indel that is stored in a variant call format file. When high-coverage whole genome sequencing is used for genotyping, $X_{i}\in \{0,1\}$ if the genotype is phased or $X_{i}\in \{0,1,2\}$ if the genotype is unphased. Otherwise, the genotype can be imputed to improve the quality of the data, leading to $X_{i}\in [0,1]$. Using genotypes and paired tissue-level expression data from individuals found in a reference transcriptomic database like GTEx [14], PrediXcan builds elastic net models for each gene based on the following formula: 


(1) \begin{align*}& \hat{y}=X \beta+\epsilon\end{align*}

where $\hat{y}$ is the estimated expression of a given gene, $\beta $ is the learned weight vector that represents how each SNV/indel affects gene expression, and $\epsilon $ is the error term. For brevity, the bias term is omitted in this review. The elastic net model fitting aims to solve the following optimization objective: 


(2) \begin{align*}& \min_{\beta} { \frac{1}{2} ||X \beta - y||_{2}^ 2 + \alpha \rho ||\beta||_{1} + \frac{\alpha(1-\rho)}{2} ||\beta||_{2}^ 2}\end{align*}

where $\alpha $ represents the penalty strength and $\rho $ represents the balance between $L1$ and $L2$ terms. While $L1$ offers desirable feature sparsity, $L2$ provides stability in learning $\beta $ when the input is perturbed.

The PrediXcan program produces a collection of per-gene elastic net models after training. These models typically focus on cis-eQTLs located near the transcriptional start sites (TSSs) ($<1$ Mb) of the genes they regulate—rather than trans-eQTLs, which act across chromosomes or from distant regions ($>1$ Mb). Although trans-eQTLs have been found to have effects on gene expression, the effect size is often much smaller than that of cis-eQTLs [3]. The trained PrediXcan models can be applied to genome-wide association study (GWAS) data, which may only include genotypes but not the respective gene expression values. The models then impute the gene expression based on the GWAS data. Finally, the imputed gene expressions are tested for association with the phenotypes of interest [3].

The idea of imputing gene expression using an additive model of genetic variants has been explored extensively in the field of eQTL analysis. Later works include: incorporating GWAS summary statistics as input [4]; extending the statistical framework to predict cross-tissue expression [5]; using Bayes methods to allow multiple tissues to share variance structure [6]; and exploring various penalty terms to improve accuracy [7]. However, PrediXcan remains one of the most popular tools.

A main differentiating factor between linear and deep learning models is how the genome sequences are represented as the model’s input. In a linear model, each feature represents a predefined SNV or indel that is found in the training data. Identifying these genetic variants often involves an elaborate process of variant calling, quality assessment, and filtering. This binary-like ($X_{i}\in \{0,1\}$ or $[0,1]$) vector representation of the genome sequence restricts its ability to generalize to previously unseen genetic variants, such as rare variants or variants derived from another cohort with a different genetic background.

Another limitation of linear models is the inability to incorporate nonlinear relationships between genotype and phenotype. If $X_{i}$ and $X_{j}$ are two variants that are located in two interacting DNA regulatory elements, there might be a synergistic effect of the pair $\{X_{i},X_{j}\}$ on gene expression. If the transcriptional machinery involves many regulatory elements in a concerted fashion, a linear model will be incapable to learn such complicated relationships.

## Deep learning-based sequence-to-function models

Recently entering the scope of computational genomics and gene expression prediction are deep learning-based sequence-to-function (S2F) models. Deep learning models can automatically learn hierarchical features directly from the data, enabling them to accept raw genome sequences as inputs, and thereby substantially increasing the flexibility of input representation. The availability of large-scale epigenomic and transcriptomic databases, such as ENCODE [15], Roadmap Epigenomics [16], 4DN [17], and GTEx [14], has enabled the development of genome AI models to predict a myriad of functional outcomes, including histone modifications, transcription factor binding, DNA accessibility, and gene expression across various tissues and cell types.

### Introduction of S2F models

**Figure 1:**
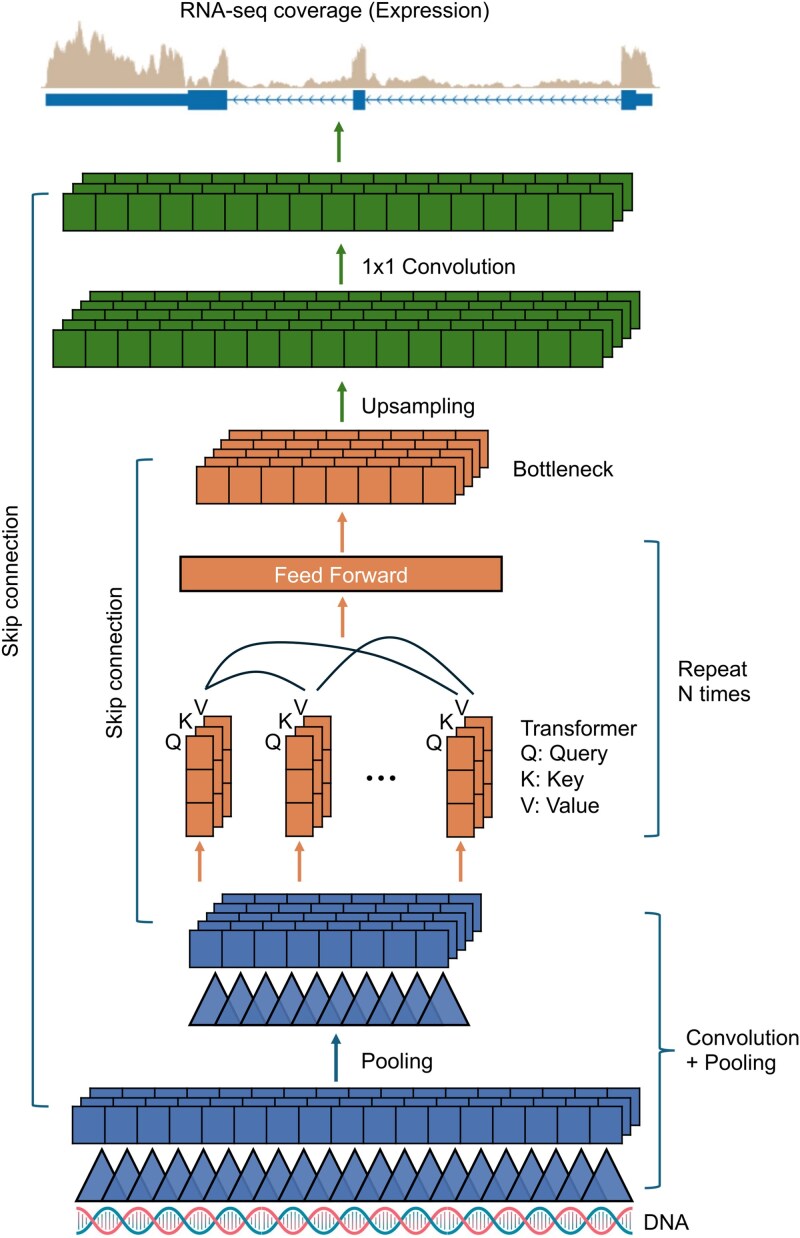
A generic deep learning-based S2F model with an encoder-decoder architecture. The model uses DNA sequences as input and can predict transcriptomic coverage at high resolution as output. The encoder consists of stacked convolution layers followed by stacked transformer layers. A convolution layer is illustrated as series of triangles that represent convolutional filters and squares that represent the activations. A transformer layer is illustrated as overlapping vectors of query (Q), key (K) and value (V) at each position, followed by a feed forward layer to produce the embeddings for the sequence. The output of the final transformer layer is a bottleneck layer that represents the final embeddings for the sequence. In some designs, the final embeddings are directly used to predict the sequence’s functional outcomes. While in others, the bottleneck layer is followed by up-sampling to increase the resolution and 1 x 1 convolution to reduce the number of channels before output

Before introducing S2F models, it is essential to outline how raw genomic information is formatted for computational analysis. Deep learning architectures typically require numerical inputs; and therefore, genomic sequences must be converted into standardized representations. A common approach is one-hot encoding, in which a sequence of length $s$ is transformed into a binary matrix $X \in \{0,1\}^{s \times 4}$. Ambiguous IUPAC nucleotide symbols (https://iupac.org/) can also be incorporated using probabilistic encodings, such as representing $N$ as $[0.25, 0.25, 0.25, 0.25]$. An S2F model can be described as the sequential application of a genome sequence encoder followed by a decoder: 


(3) \begin{align*}& \hat{y} = f(g(X))\end{align*}

where $g$ is a sequence encoder which produces a representation (i.e. embedding) of the sequence; $f$ is a decoder that uses the embedding to predict the various functional outcomes for different tissues and cell types; $\hat{y}\in \mathbb{R}^{t\times d}$ represents the output data tracks across $t$ bins and $d$ molecular phenotypes. The decoder $f$ can be a simple linear layer or a sequence of stacked up-sampling and 1 × 1 convolution or up-convolution layers. Often times, $t < s,$ which indicates the reduction in resolution by a deep learning model thereby each bin represents the average value of $s/t$ base-pairs. Depending on the design of decoder $f$, $t$ can also be the same as $s$, which means no loss in resolution nevertheless incurs more computation in the decoder. $d$ is typically in the order of thousands to represent different epigenomic events and gene expression across hundreds of tissues and cell types. The function $g$ can be a neural network with a sophisticated architecture to reflect the complicated nature of transcriptional regulation. For example, 1D convolution layers are used to detect transcription factor binding motifs and their combinations; transformer layers [18] are used to detect long-range interactions among DNA regulatory elements. Figure 1 illustrates the idea of an S2F model. In principle, these models can accept arbitrary genomic sequences as input, with the maximum feasible sequence length $s$ limited only by available computational resources. Because the training and evaluation sequences need not overlap, the models are capable of processing inputs that contain novel genetic variants not observed during training.

Convolution and transformer layers are the key neural network elements that compose an S2F model for sequence-to-expression prediction. A convolution or transformer layer is controlled by different parameters and can be enhanced by techniques such as skip connection. These design choices can greatly affect a model’s performance and computational cost. Here are some of the most important factors in model design:

- **Convolution:** A convolutional layer applies a set of learnable filters across local regions of the input to extract informative features. Because the same filter is applied at every position, convolutional layers efficiently capture local patterns while providing a degree of translational invariance. In most genomics applications, kernel size and number of filters are the two most influential design parameters. A larger kernel captures a broader spatial context (i.e. expands the receptive field), but it increases the number of weights and the computational cost linearly. Each filter acts as a specialized feature detector, so increasing the number of filters generally improves the model’s capacity to learn diverse patterns; however, doing so also raises the computational cost and can increase the risk of overfitting.
- **Dilation:** Dilation introduces gaps between the elements of a convolutional kernel, expanding its effective receptive field without increasing computational cost. However, large dilation rates can cause the model to miss fine-scale details and may introduce “gridding” artifacts.
- **Transformer:** A transformer layer is built around self-attention, a mechanism that allows each position in a sequence to weight and integrate information from all other positions. This enables transformers to model long-range dependencies more effectively than convolutional or recurrent architectures. The most influential parameters include the embedding dimension, the number of attention heads, and the feed-forward network (FFN) width. Increasing the embedding dimension or FFN width expands the model’s representational capacity, but it also increases computational cost and memory usage, with the self-attention operation scaling quadratically as $O(L^{2})$. The number of attention heads controls how many distinct relation patterns the model can attend to in parallel; while more heads can enrich feature learning but add parameters and runtime.
- **Up-sampling and 1 x 1 convolution:** Up-sampling layers are used in the decoder to progressively restore spatial or sequential resolution after the encoder compresses it through pooling or strided convolutions. Up-sampling can be performed in several ways—including interpolation, up-convolution (i.e. transposed convolution), and the simple repetition of values—to increase resolution before refining the features with subsequent convolutions. A “1 $\times $ 1” convolution is a conventional term originating from 2D image processing; in genomics applications, the analogous operation is a 1D convolution with a kernel size of 1, which mixes information across channels without changing the sequence length. This operation is commonly applied when merging up-sampled decoder features with encoder skip connections, allowing for flexible channel adjustment and efficient feature fusion. Together, up-sampling and 1D “1 $\times $ 1” convolutions enable the network to recover fine-grained structure while maintaining computational efficiency.
- **Skip connection:** Skip connections directly pass features from earlier layers to later layers, bypassing intermediate operations. In encoder–decoder architectures, they connect encoder feature maps to corresponding decoder layers at matching resolutions. This provides the decoder with high-resolution, fine-grained information that would otherwise be lost through pooling or downsampling, improving localization and detail recovery. More generally, skip connections help gradients flow more easily during training, mitigating vanishing gradient issues and enabling deeper networks to learn effectively.

Figure 1 illustrates an encoder–decoder architecture used for an S2F model. Although higher resolution and longer context windows are desirable for capturing fine-grained regulatory signals, they substantially increase computational demands and model complexity, which can in turn raise the risk of overfitting. Designing an S2F model for sequence-to-expression prediction therefore becomes a careful balance between efficiency and representational power. Choosing appropriate convolutional and transformer parameters—such as kernel size, number of filters, embedding dimension, and attention heads—plays a central role in navigating this tradeoff and tailoring the model to the available data and computational budget.

### Overview of selected S2F models

DeepSEA [19] is a pioneering model that first demonstrated the ability of deep learning to predict an array of functional genomic annotations, including transcription factor binding, DNase I hypersensitivity, and histone modifications, solely from a DNA sequence. Utilizing a three-layer convolutional neural network (CNN) trained on 1000 bp inputs, DeepSEA established the S2F prediction paradigm. Importantly, while revolutionary, this initial architecture was a small-scale network by modern standards, outputting only one prediction value per chromatin feature for the entire 1000 bp region, thus lacking explicit spatial information within the input. Despite this limitation, DeepSEA served as a crucial proof-of-concept, laying the foundation for quantifying the impact of noncoding variants on chromatin features and launching the field of S2F prediction.

Subsequent S2F models introduced architectural and design variations to manage the inherent trade-offs among output feature richness, resolution, context length, and computational efficiency. For instance, ExPecto [20] built upon the DeepSEA foundation by significantly enhancing the network’s capacity: it doubled the number of convolutional layers, increased the number of predicted tissues and cell types, and expanded the direct input context window to 2000 bp. Critically, ExPecto introduced a spatial feature integration layer that aggregates predictions within a 40-kb TSS-centered context to ultimately predict gene expression in specific tissues. While these improvements substantially enhanced model capability, particularly for expression prediction, the approach was still constrained by a relatively short effective context window (40 kb) for gene expression and a medium resolution (200 bp) for chromatin features.

Basenji2 [21] expanded the input context dramatically to 131 kb, using deep stacks of dilated convolutions to enlarge the model’s receptive field and capture long-range regulatory interactions across genomic regions. This broader context substantially improved predictive performance, with Basenji2 achieving a Pearson correlation of 0.81 for gene expression prediction. However, the expanded architecture also increased the number of trainable parameters, resulting in a larger memory footprint and greater computational cost. Moreover, although dilated convolutions extend the range of dependencies that can be modeled, they inherently specialize in detecting local patterns and lack explicit global integration mechanisms. As a result, Basenji2 is still limited in its ability to capture ultra-long-range chromatin interactions and genome-wide regulatory context.

Xpresso [22] is a lightweight sequence-to-expression model designed to predict steady-state mRNA abundance directly from a promoter sequence alone. It combines a shallow 2-layer CNN with hand-engineered features reflecting mRNA stability—such as GC content, UTR length, and coding exon density—to capture both transcriptional and post-transcriptional determinants of gene expression. By integrating these biologically informed features in a 10.5-kb window with a compact CNN architecture, Xpresso achieved strong performance with far fewer parameters than deep regulatory models, explaining up to 60%–70% of the variance in mRNA levels across human and mouse genes. Its simplicity also makes it computationally efficient and easily interpretable, enabling insights into promoter architecture and sequence determinants of expression. However, Xpresso’s reliance on the promoter sequence and a fixed set of stability features limits its ability to model the rich landscape of distal regulation, chromatin state, enhancer–promoter interactions, and context-specific regulatory logic. As a result, while it performs well for genes whose expression is largely promoter-driven, it is less effective for genes governed by long-range or chromatin-mediated regulatory mechanisms, highlighting the need for architectures capable of integrating a broader genomic context.

A central challenge that emerges from these models is the difficulty of capturing long-range regulatory interactions that underlie gene expression. While ExPecto made early steps toward addressing this by introducing a spatial integration layer that aggregates predicted chromatin features across a 40-kb window around each TSS, this mechanism remains relatively coarse and limited in its ability to model the complex, nonlinear interplay among distal enhancers, promoters, and chromatin architecture. Basenji2 pushed the field further by dramatically expanding the input context and employing dilated convolutions to propagate information across 131 kb. However, dilated convolutions are fundamentally local operators: although they extend the receptive field, they lack explicit mechanisms for global interaction modeling and struggle to integrate signals across hundreds of kilobases or megabase scales. Together, these limitations highlight a major bottleneck in sequence-to-expression modeling—effectively learning regulatory interactions that span vast genomic distances—and motivate the development of architectures capable of true long-range reasoning, such as transformer-based models.

Enformer [23] represents a major breakthrough in sequence-to-function modeling, introducing a hybrid architecture that combines convolutional layers with transformer blocks to capture long-range regulatory interactions through self-attention. Whereas convolutional models are fundamentally limited by their finite receptive fields, the transformer layer enables Enformer to integrate information across the entire input window and model interactions between distant genomic elements. This directly addresses a key limitation of earlier S2F models, whose top-layer receptive fields remained modest—approximately $\pm 20$ kb for ExPecto, $\pm 27.5$ kb for Basenji2, and only 7 kb upstream and 3.5 kb downstream for Xpresso. In contrast, Enformer achieves a receptive field spanning $\pm 98.3$ kb, allowing it to “see” roughly 84% of high-confidence enhancer–gene pairs, compared with just 47% for the previous state-of-the-art Basenji2. This expanded context yields substantial improvements in predictive accuracy, increasing the Pearson correlation for CAGE-based gene expression prediction to 0.85.

Building on Enformer, Borzoi [24] further extends the context window to 524 kb while increasing the spatial resolution to 32 bp using a U-Net–like architecture [25]. Unlike Enformer, which applies linear layers at the bottleneck to generate predictions, Borzoi incorporates a decoder that progressively up-samples feature maps to recover high-resolution chromatin profiles. The recently introduced AlphaGenome [26] adopts a similar encoder–decoder design but expands the receptive field to 1 Mb and achieves single–base-pair resolution. In both Borzoi and AlphaGenome, U-Net–style decoders employ 1 $\times \ $1 convolutions to modulate channel depth during up-sampling and use skip connections to fuse encoder and decoder representations while preserving spatial detail. These advances greatly enhance the complexity of functional outputs and improve predictive accuracy; however, they come with substantial computational costs—e.g. AlphaGenome training required hundreds of TPUv3 chips and dozens of NVIDIA H100 GPUs.

Table 1 summarizes the models discussed above, illustrating the progression from DeepSEA to AlphaGenome as context windows expand and spatial resolution increases. In S2F architectures, model design inevitably involves negotiating trade-offs among four primary factors: the breadth of output features representing tissue- and cell type-specific functional readouts; the context length required to capture long-range regulatory interactions; the spatial resolution necessary for variant-level and mechanistic interpretation; and the computational cost, which escalates rapidly with model capacity.

**Table 1:** Summary of S2F models that use genome sequence as input to predict functional outcomes. All models were evaluated across genomic regions

Model	Year	Modeling approach	Context window	Resolution	Performance highlights
DeepSEA [19]	2015	Three-layer CNN	1 kb	1 kb	Median AUC 0.86–0.96 for various chromatin features
ExPecto [20]	2018	Two-stage model: CNN + exponential basis linear model	40 kb around TSS	200 bp	0.82 Spearman correlation on tissue-specific expression prediction
Basenji2 [21]	2020	Deep residual CNN (dilated conv layers)	131,072 bp	128 bp	Pearson $ R = 0.81 $ for gene expression (CAGE signal at TSS)
Xpresso [22]	2020	Shallow CNN on promoter sequence (+ mRNA stability features)	10.5-kb promoter region	Gene-level output	$ R^{2} = 0.59 $ (human) and 0.71 (mouse)
Enformer [23]	2021	Hybrid CNN–Transformer	196 kb	128 bp	Pearson $ R = 0.85 $ for gene expression (CAGE across genes)
Borzoi [24]	2025	CNN + Transformer (Enformer-based architecture with upsampling for fine resolution)	524 kb	32 bp	Pearson $ R = 0.87 $ for gene-level expression
AlphaGenome [26]	2025	Deep CNN + Transformers (Borzoi-like architecture with sequence parallelization)	1 Mb	1 bp	State-of-the-art performance (e.g. 17.4% improvement in gene expression prediction versus Borzoi)

Despite these architectural advances, all existing deep learning models are trained on the reference genome, using annotations sourced from reference databases such as ENCODE [15] and FANTOM [27]. Consequently, it remains unclear to what extent the regulatory mechanisms learned from this single genomic background can generalize to explain personal variation in gene expression.

### Using S2F models for personal gene expression prediction

The high accuracy of S2F models in predicting gene expression and other molecular phenotypes across genomic regions can be attributed to a neural network’s ability to learn the transcriptional regulation language using genome sequence alone. This has raised the question whether they can be used to interpret genetic variations between individuals. A recent study [12] compares four models—ExPecto, Xpresso, Basenji2, and Enformer on the Geuvadis consortium [28] dataset to address this question. The key benchmark statistics of the study are summarized in Table 2. They found that these models achieved relatively high correlation values when tested across genes in a single individual. This can be attributed to their ability to learn generalizable cis-regulatory rules from sequence, such as transcription factor binding sites, which remain largely consistent across genes within an individual genome. However, the effects of individual-specific genetic variants on gene expression remain elusive, even when the same gene is tested. Because these models are not trained on individual-level genetic variation, they learn cis-regulatory rules although do not account for how specific genetic differences in individual genomes impact gene expression. As a result, all deep learning models achieved drastically lower correlation values during cross-individual testing: even the highest performing Enformer achieved a Spearman correlation of only 0.045 on the Geuvadis consortium dataset. While PrediXcan when trained and evaluated on the same dataset can achieve a Spearman correlation of approximately 0.25.

**Table 2:** Spearman rank correlations for different gene expression prediction tasks on the Geuvadis consortium dataset

Task	Input	Target	Enformer	Basenji2	ExPecto	Xpresso	PrediXcan
Reference correlation	Reference sequence	Median expression	0.57	0.52	0.53	0.33	–
Across genes	Individual sequence	Individual expression	0.55	0.51	0.52	0.32	–
Across individuals	Individual sequence	Individual expression	0.045	0.037	0.034	0.007	0.25

A similar study with the same goal draws the same conclusion on a different dataset [11]. They found that PrediXcan effectively modeled gene expression across individuals, significantly predicting 921 of 13,397 protein-coding genes with a mean Pearson correlation of 0.26 when tested on the ROSMAP [29] dataset, while Enformer only predicted 162 genes and obtained a mean Pearson correlation of only 0.02. This benchmark study also indicates that Enformer may over-rely on short-range DNA motifs, focusing primarily on promoter sequences near the TSS rather than on distant enhancers. Furthermore, Enformer mis-attributed the effects of variants, assigning too much importance to the wrong SNVs, leading to mis-predictions. Finally, many SNVs flagged by Enformer as important did not affect gene expression in real eQTL datasets. These unsupported driver SNVs contributed to Enformer’s lack of effectiveness, and even when Enformer identified potential driver variants, it often mis-predicted the direction of their effect on gene expression. Enformer’s problems with directionality may stem from its limited understanding of cis-regulatory variants. If an enhancer normally activates a gene yet is mutated in a way that disrupts activation, then gene expression should decrease. However, if Enformer ignores the enhancer and instead focuses on a promoter SNV that has little impact on gene expression, it may inaccurately infer that the expression should increase.

Several factors likely contribute to the inability of S2F models to predict personalized gene expression. First, all of the S2F models discussed above are trained exclusively on the reference genome, which increases the risk of overfitting and limits their ability to generalize to individual genomes. Second, personal genomes often contain rare variants and structural variants that are absent from the reference genome, further reducing model accuracy. Third, S2F models still struggle to capture long-range regulatory interactions, even when transformer architectures are used. For example, a recent study [30] reported that Enformer failed to predict the functional effects of enhancers located far from the TSS; its ability to explain expression variance for enhancer perturbations 30 kb away from the TSS was nearly negligible. It remains unclear whether this limitation arises from the model’s difficulty in recognizing regulatory elements, identifying enhancer–promoter pairings, or both.

### Fine-tuning S2F models

Due to these shortcomings, researchers have been attempting to finetune S2F models and develop variant-aware sequence-to-expression models to improve cross-individual predictions. Due to the high cost of sample collection and sequencing, datasets with paired whole genome sequencing and RNA-seq are scarce and limited in sample size. Table 3 lists the datasets for the evaluation and finetuning of personal expression prediction models reviewed in this study.

**Table 3:** List of datasets with paired whole genome sequencing and gene expression data

Dataset name	Tissue/Cell type	*N* donors
GTEx [14]	54 tissue types such as uterus, ovary, adipose, stomach, and lung	838
ROSMAP [29]	Post-mortem brain tissues, with the major region being the dorsolateral prefrontal cortex (DLPFC)	1894 for DLPFC
Geuvadis [28]	Lymphoblastoid cell lines	462

Performer [31] is one of the first studies to finetune the Enformer model on paired WGS and RNA-seq samples for personal gene expression prediction. Focusing on the “whole blood” tissue from the GTEx database [14], Performer selected 300 genes that span a range of cis-heritability and used the individual genome sequences from 670 subjects to train and evaluate Enformer for the tissue-specific gene expression prediction. To improve the model’s ability to capture the variation across individuals, Performer’s loss function includes a component that looks at the pairwise difference between each pair of individuals within a batch. For a pair of individuals $\{i,j\}$: if the difference between observed values is $(o_{i}-o_{j})$ and predicted values is $(p_{i}-p_{j})$, Performer minimizes the difference between the two thereby $[(o_{i}-o_{j})-(p_{i}-p_{j})]^{2}$ for all pairs $\{i,j\}$ are included in its loss. While Enformer’s predictions often show little or even negative correlations across the GTEx individuals, the Performer corrects this for most genes, showing performance that are as good as or even better than those of elastic nets for 84% of the genes. They also trained Performer to predict DLPFC expression in the ROSMAP dataset with 742 individuals and made predictions for 205 GTEx individuals with brain cortex expression available, and observed good cross-cohort performance.

The Performer study attempts to train a model for 300 genes jointly to predict personal gene expression, however does not find any improvement against a single-gene model. When the multi-gene Performer model is used on held-out genes whose individual-level expression was not used to train the model, it can explain only the same amount of variability as the untrained Enformer model. These experiments suggest that the transcriptional regulatory mechanisms of cross-gene and cross-individual contexts speak fundamentally different languages, with little overlap between them. The results provide additional evidence to explain Enformer’s poor performance in predicting personal expression in the studies mentioned above [11, 12]. Additional experiments include using finetuned Borzoi for personal gene expression prediction although find it to perform similarly to Performer, indicating little improvement from a different model architecture.

In a separate study [32], researchers draw the same conclusion when they finetune Enformer on a lymphoblastoid cell line gene expression dataset with 421 individuals from the Geuvadis consortium. The finetuned deep learning model performs similarly to a linear model trained on the same data. Additional training strategies are tested. To avoid a phenomenon known as catastrophic forgetting [33], they jointly train Enformer on both the novel personalized expression dataset and its original training dataset. To augment the genetic variation seen by the model beyond the relatively small personalized expression dataset, they use a Massively Parallel Reporter Assay (MPRA) data in addition to the personal expression dataset. However, neither training strategy leads to significant improvement. The Geuvadis data are based on individuals with European ancestry [28]. To further evaluate the models, they are used to make predictions on a population with Yoruban ancestry. Pearson correlations drop by more than 0.1 for all models tested, indicating the difficulty in generalizing predictive models to another group of individuals with a different genetic background.

## Genomic language models

Inspired by advances in natural language processing (NLP), recent efforts have developed genomic language models (gLMs) that treat DNA sequences as structured text, applying transformer-based architectures or state space models (SSMs) to learn rich contextualized representations of genomic information [34]. Models such as DNABERT-2 [35], Nucleotide Transformer (NT) [36], and Evo 2 [37] are pretrained on large-scale, unlabeled genomic corpora using self-supervised objectives such as masked language modeling (MLM) or autoregressive (AR) language modeling. These models capture the statistical structure and “syntax” of the genome, including regulatory motifs, sequence composition, and long-range dependencies. Unlike supervised models trained for specific tasks, gLMs produce general-purpose embeddings that can be fine-tuned or directly applied to a broad range of downstream applications, including promoter classification, enhancer activity prediction, transcription factor binding site identification, and non-coding variant effect inference. Their ability to transfer learned representations across tasks, cell types, and even species positions them as foundation models for scalable, data-efficient genome interpretation, marking a significant shift toward universal pretraining in genomics. Creating a gLM involves several key tasks that parallel those in NLP nevertheless require genomic-specific adaptations. They are described in the following.

### Corpus construction

The first step in developing a gLM involves constructing a suitable training corpus. This typically entails selecting one or more reference genomes—such as human, mouse, or a multi-species compendium—to provide a broad and representative sampling of genomic content. Depending on the model’s intended application, the corpus may include only specific regions (e.g. promoters or introns) or encompass the entire genome, including coding, non-coding, and repetitive elements. These genomes are then segmented into fixed-length sequence windows (e.g. 1 kb to 1 Mb), which serve as input samples for model training.

### Tokenization

Unlike natural language, DNA consists of a small four-letter alphabet (A, C, G, T). While representing individual nucleotides as tokens has been explored, most approaches employ specialized tokenization strategies to capture more expressive and informative representations of sequence content. Three widely used strategies are:

- **k-mer tokenization:** This is the most common approach in gLMs. A DNA sequence is parsed into fixed-length subsequences (k-mers), such as 3-mers or 6-mers. Models like DNABERT [38] use overlapping k-mers (stride = 1), preserving high-resolution positional information and capturing local motifs. In contrast, models like the NT employ non-overlapping k-mers (stride = k) to reduce the number of tokens per input, enabling efficient training over long genomic contexts (e.g. kilobases). The choice of k introduces a key trade-off: larger k captures longer sequence motifs yet exponentially increases the vocabulary size (e.g. $4^{6} = 4,096$), which raises memory and computational costs. At the same time, using a larger k reduces the total number of tokens per input sequence, which can improve scalability by lowering the quadratic cost of self-attention. Selecting an optimal k thus balances resolution, vocabulary complexity, and sequence length.
- **Byte pair encoding (BPE) and subword tokenization:** Originally developed for NLP, BPE, and other subword-based tokenization schemes have recently been adapted to genomics to create more flexible and data-driven vocabularies. These methods iteratively merge frequent character or k-mer pairs to form longer, biologically meaningful tokens without fixing k in advance. This can result in smaller, more compact vocabularies and greater expressiveness, especially for handling diverse or repetitive sequence patterns. While not as widely used as k-mer tokenization, BPE has been explored in models such as DNABERT-2 and GROVER [39], offering a promising alternative for capturing variable-length sequence motifs.
- **Nucleotide tokenization:** It treats each base (A, C, G, T) as an individual token rather than grouping them into k-mers. This approach preserves the full base-level resolution of the genome, however substantially increases sequence length, posing challenges for transformer architectures with quadratic scaling in sequence length. Consequently, nucleotide-level tokenization is more commonly employed in SSMs with linear-time complexity, which can efficiently process very long genomic sequences while retaining nucleotide-level detail.

### Pretraining objective

Pretraining gLMs relies on self-supervised learning objectives to extract meaningful representations from unlabeled DNA sequences. Two primary formulations are widely used:

- Masked language modeling: MLM is a bidirectional objective, originally introduced in BERT [40], where the model is trained to predict a randomly masked subset of tokens from their surrounding context. Let a DNA sequence be represented as a tokenized input $ \mathbf{x} = (x_{1}, x_{2}, \dots , x_{T}) $, and let $ \mathcal{M} \subset \{1, \dots , T\} $ denote the indices of tokens that are masked. The objective is to minimize the expected negative log-likelihood over the masked positions:
  \begin{align*} & \mathcal{L}_{\mathrm{MLM}} = \mathbb{E}_{\mathcal{M}} \left[ - \sum_{i \in \mathcal{M}} \log P(x_{i} \mid \mathbf{x}_{\backslash \mathcal{M}}) \right], \end{align*}
  where $ \mathbf{x}_{\backslash \mathcal{M}} $ denotes the input sequence with the masked tokens replaced (e.g. with a [MASK] token). This formulation enables the model to learn from both upstream and downstream context, making it well-suited for genomic sequences where regulatory signals are often non-directional. MLM is used in models such as DNABERT, DNABERT-2, and NT. One limitation of MLM is the *pretrain–finetune mismatch*, since masked tokens may not appear during downstream inference.
- Autoregressive language modeling: In contrast, AR models are trained to predict the next token in a sequence using only the left context. For a sequence $ \mathbf{x} = (x_{1}, x_{2}, \dots , x_{T}) $, the objective is to minimize the negative log-likelihood of each token conditioned on its preceding tokens:
  \begin{align*} & \mathcal{L}_{\mathrm{AR}} = - \sum_{t=1}^{T} \log P(x_{t} \mid x_{1}, x_{2}, \dots, x_{t-1}). \end{align*}
  This unidirectional formulation is used in models inspired by GPTs [41–43] and is especially suitable for generative tasks, such as genomic sequence synthesis. AR models avoid the use of artificial masking tokens and preserve the full input distribution during training. Recent gLMs such as Evo 2 and DNAGPT [44] employ AR objectives to learn from long, contiguous DNA sequences.
  Although AR models naturally lack bidirectional context, recent work has begun to address this limitation. For example, Caduceus [45] introduces a novel bidirectional technique by applying a model to both a sequence and its flipped version and then join information from the forward and backward copies. These innovations blur the line between traditional AR and MLM approaches, enabling richer contextual learning without sacrificing generative capability. While AR models may still be less intuitive for tasks requiring fully bidirectional context, they show strong promise for modeling long-range dependencies and supporting both discriminative and generative applications in genome-scale settings.

### Model architecture

Earlier works used recurrent neural networks (RNNs) [46] and CNNs [47] to capture local and short-range dependencies in genomic sequences. Most gLMs today adopt transformer-based architectures because of their ability to model long-range interactions through self-attention [18]. However, transformers scale quadratically with sequence length ($ \mathcal{O}(L^{2}) $), making them computationally prohibitive for modeling megabase-scale genomic contexts. Recent developments [48] have introduced SSMs as an efficient alternative for modeling long genomic sequences when scalability is essential.

SSMs replace pairwise attention with linear recurrent updates governed by continuous or discrete dynamical systems. In a simplified discrete-time formulation, an SSM maintains a hidden state $ \mathbf{h}_{t} \in \mathbb{R}^{N} $ that evolves linearly with the input sequence $ x_{t} $: 


\begin{align*} & \mathbf{h}_{t+1} = \mathbf{A} \mathbf{h}_{t} + \mathbf{B} x_{t}, \quad y_{t} = \mathbf{C} \mathbf{h}_{t}, \end{align*}

where $ \mathbf{A} $, $ \mathbf{B} $, and $ \mathbf{C} $ are learnable parameters that define the system’s dynamics, input projection, and output mapping, respectively. Because this recurrence is linear in both $ \mathbf{h}_{t} $ and $ x_{t} $, the entire sequence can be expressed as a convolutional operation: 


\begin{align*} & y_{t} = \sum_{k=0}^{t} K_{k} \, x_{t-k}, \quad \mathrm{where}\ K_{k} = \mathbf{C} \mathbf{A}^{k} \mathbf{B}. \end{align*}

This kernel formulation allows efficient computation using fast Fourier transforms, yielding linear time and memory scaling ($ \mathcal{O}(L) $) with respect to sequence length. Importantly, the recurrence matrix $ \mathbf{A} $ defines a long-range memory kernel whose eigenstructure determines how information decays or persists over time. Properly parameterized (e.g. via diagonalization [49] or HiPPO initialization [50]), $ \mathbf{A} $ can retain stable, slowly decaying modes, allowing SSMs to preserve information across hundreds of thousands of tokens—a key advantage over conventional RNNs.

Intuitively, SSMs behave like learnable filters that integrate information over arbitrary ranges, combining the efficiency of convolutions with the sequential structure of dynamical systems. This makes them well-suited for genomic modeling, where distal enhancer–promoter or chromatin-level interactions may span hundreds of kilobases. Recent genomic architectures such as HyenaDNA [48] and Evo 2 [37] leverage these properties to pretrain over megabase-scale inputs, achieving both long-range expressivity and computational tractability.

### Overview of selected gLMs for personal gene expression prediction

Since 2020, the field has experienced a rapid expansion in the development of gLMs. Although a comprehensive review of these models is beyond the scope of this study, readers are referred to recent reviews [51] that provide in-depth discussions of gLM advances. Table 4 lists the gLMs that are reviewed in this study.

**Table 4:** Overview of the gLMs used for personal gene expression prediction

Model name	Sequence corpus	Context	Pretraining	Architecture
Nucleotide Transformer [36]	Human reference, 3200 diverse human genomes, and 850 genomes of various species	6000 bp	MLM	Transformer
Nucleotide Transformer v2 [36]	Same as NT	12 kb	MLM	Transformer
DNABERT-2 [35]	Human reference and 135 species: 32.5 billion nucleotides total	10 kb	MLM	Transformer
UKBioBert [52]	Synthetic sequences created by inserting 13 million SNPs from UKBioBank into human reference	100 kb	MLM	Transformer
Caduceus [45]	Human reference genome	1 Mb	AR	SSM
Evo 2 [37]	OpenGenome2: a database of 8.84 trillion nucleotides of curated DNA from diverse species	1 Mb	AR	SSM

NT is one of the first large-scale gLMs, which is trained on 850 genomes across multiple species and 3200 human genomes. It utilized MLM on 6-kb DNA sequence segments that were segregated into 1000 non-overlapping 6-mer tokens. In extensive evaluations across 18 downstream genomic tasks—ranging from promoter prediction to splice site classification and epigenetic state inference—the NT consistently outperformed existing models. In a recent study [53], the authors employed NT to generate embeddings from 4-kb regions centered on the TSS for 290 GTEx participants, aiming to exploit its ability to encode inter-individual genetic variation. These embeddings were then kept fixed while additional transformer layers were trained for personalized gene expression prediction. However, this approach still fell short when compared to linear models, with the cross-individual median Pearson correlation being 0.02 less than that of Elastic Net. This shows that the pretraining of a transformer network on thousands of genomes, and the additional training of transformer layers on a small-size personal expression dataset failed to capture the individual transcriptional regulation. Furthermore, its performance deteriorated significantly as gene length increases-showing a negative correlation ($ P = 1.9 \times 10^{-6} $), which indicates the difficulty to learn long-range interactions for personal expression prediction even with a relatively small context window. Finally, its sequence input length of 4 kb is notably lower than that of Enformer (197 kb), potentially preventing the modeling of regulatory effects that could influence gene expression. NT-v2 is the second generation of gLM in the NT family, focusing on more efficient network architecture that reduces the number of parameters. Consequently, it increases the context window from 6 to 12 kb. In a recent study [54] that compares several gLMs’ performance in personal gene expression using the Geuvadis data, NT-v2 was found to be similar in performance to Evo 2 although underperformed another gLM called Caduceus. Furthermore, it still performed worse than Elastic Net.

DNABERT-2 is another notable gLM that builds on the original DNABERT by introducing several techniques to improve computational efficiency and effectiveness, and shows stronger empirical performance on genomic tasks. Unlike its predecessor, DNABERT-2 uses BPE as tokenization and attention with linear bias for positional embedding, and is pretrained on a broader genomic corpus. In direct comparisons with the NT, DNABERT-2 demonstrates comparable or superior performance on several core tasks despite being significantly smaller. In a recent study [52] designed to extend the DNABERT-2 framework, the authors introduced UKBioBert, a model pretrained on synthetic genomic sequences generated by incorporating 13 million genetic variants—derived from approximately 300,000 individuals—into the reference genome. This variant-informed pretraining strategy enables the model to better capture patterns of individual genetic variation within an MLM framework. The embeddings produced by UKBioBert were then integrated with the embeddings from Enformer and finetuned for personal expression prediction. The resulting model, known as UKBioFormer, fuses Enformer’s sequence-to-expression features with UKBioBert’s variant-aware sequence representations into a single architecture. By doing this, UKBioFormer enhances Enformer, outperforming Performer in 63.3% of genes with good predictability (Pearson correlation > 0.6 in Europeans) during cross-cohort testing (European to African American). Additionally, in predicting the direction of eQTLs, a task Enformer greatly struggles with, UKBioFormer achieved an accuracy of >70%, while Enformer only achieved an accuracy of 53% and Performer achieves an accuracy of 68%. However, like other deep learning models, UKBioFormer still struggles to generalize well to genes it was not fine-tuned on.

Caduceus and Evo 2 represent two recent SSM architectures that extend the capabilities of gLMs to megabase-scale contexts. Both models are descendants of the broader SSM family; however, differ in their architectural foundations and biological inductive biases. Caduceus builds on the Mamba framework [55], a selective SSM designed for long-sequence processing with near-linear complexity ($ \mathcal{O}(L \log L) $). What makes Caduceus unique among gLMs is its incorporation of domain-specific inductive biases. It extends the Mamba block into two specialized variants: BiMamba, which introduces bi-directional processing to capture both upstream and downstream dependencies, and MambaDNA, which enforces reverse-complement (RC) equivariance to respect the double-stranded symmetry of DNA. These adaptations enable Caduceus to model sequence context in both directions and to generalize robustly across complementary strands—properties critical for genomic tasks such as variant effect prediction and motif recognition. Evo 2 adopts a different SSM-inspired design, based on the StripedHyena 2 architecture [56], the first multi-hybrid model built upon input-dependent convolutions to efficiently capture short-, medium-, and long-range dependencies across sequences. Evo 2 leverages this framework to train autoregressively on over 9 trillion nucleotides from all domains of life including Bacteria, Archaea, Eukaryota, and Viruses, enabling single-nucleotide resolution modeling at genome-scale context lengths.

Thus, while both models share the efficiency and long-context advantages of the SSM family, Caduceus emphasizes biologically grounded inductive biases—such as bi-directionality and RC symmetry—whereas Evo 2 focuses on large-scale sequence corpus, species diversity, and multi-operator flexibility for universal genomic modeling. In a recent benchmark [54] comparing multiple deep learning models for personalized gene expression prediction, Caduceus not only outperformed all gLMs, including Evo 2, yet also surpassed S2F architectures, achieving a mean Spearman correlation of 0.20 compared to 0.11 for the best S2F model. Moreover, across a subset of 46 genes deemed unpredictable by Elastic Net, Caduceus achieved a mean Spearman correlation of 0.11. Notably, these results were obtained despite Caduceus being pretrained solely on the human reference genome, whereas Evo 2 was trained on a massive cross-species corpus. This contrast underscores that architectural innovations inspired by biological principles can yield substantial improvements in modeling and interpreting transcriptional regulation from genomic sequences.

## Discussion and future directions

The development of genome AI models using large-scale reference genomic and epigenomic databases has opened a new avenue for researchers to study a decades-old problem: the relationship between human genetic variants and health and disease. However, the direct application of these models to personal genomes has failed to achieve reliable predictive accuracy, yielding low or even negative correlations across certain genes. One possible explanation is that the sequences governing transcriptional regulation among genes encode mechanisms fundamentally different from those of genetic variants among individuals. To address this, researchers finetuned the models on datasets containing paired genome and gene expression data from cohorts of individuals. The resulting models showed improvements over the original versions, reversing most of the negative correlations, however did not outperform linear models significantly. This may be due to the relatively small size of the finetuning datasets—which typically include only hundreds of individuals—being insufficient for deep learning models to generalize well to unseen samples. It is also possible that novel network architectures and loss functions are needed for more effective training in this specific context.

The introduction of gLMs has opened the door to new possibilities. Because the pretraining relies solely on genome sequences, a gLM can leverage the vast number of personal genomes that have been sequenced and deposited into public databases. This circumvents the need for paired genomic and epigenomic data from each individual to train a supervised model—data that are often prohibitively expensive and time-consuming to collect. Ideally, such pretrained models require only a small number of additional samples for downstream tasks, thereby significantly improving data efficiency. In the field of large language models (LLMs), the scaling law [57] consistently holds true: more training data, larger models, and greater computational power tend to yield better performance. Today’s state-of-the-art LLMs required enormous computational resources to train, with estimated costs exceeding 100 million US dollars [58]. It is reasonable to speculate that a similar scaling law may apply to gLMs. However, current leading models such as NT were trained on only thousands of personal genomes. Meanwhile, UKBioBERT was trained on synthetically mutated sequences rather than actual personal genomes due to UK Biobank’s privacy regulations. Evo 2 is heading towards that direction by utilizing a sequence corpus with nearly nine trillion nucleotides. These gLMs are still nowhere near the scale of LLMs like GPT-4 [59] or Grok 4 [60]. Whether a GPT-like gLM holds the key to unlocking personal expression prediction remains an open question.

Looking ahead, the next generation of genome AI models will depend critically on the creation of larger and more diverse individual-level datasets. Current models are predominantly trained on reference genomes or limited population panels, which constrains their ability to capture the full spectrum of human regulatory and genetic variation. Building datasets that couple genomic sequences with transcriptomic, epigenomic, proteomic, and phenotypic measurements across diverse ancestries, tissues, and environmental contexts will be essential to advance from generic to individualized genome modeling.

Beyond data collection, there is an urgent need for methodological breakthroughs that go beyond scaling and instead embrace the complexity of biology. The strong performance of Caduceus on the Geuvadis dataset highlights the importance of incorporating biological insights into model design. This finding suggests that merely increasing model size or training data may yield diminishing returns unless existing biological knowledge is leveraged to guide models toward extracting meaningful regulatory signals from genomic sequences. Future models should therefore pursue architectures, objectives, and pretraining strategies that explicitly encode principles such as allelic heterogeneity, epistasis, and context-specific regulation. Multi-omics integration represents a powerful avenue toward this goal: by learning joint representations across DNA, RNA, and chromatin modalities, models could more directly link sequence variation to molecular and cellular phenotypes. The General Expression Transformer study [61] demonstrated that integrating DNA accessibility with sequence information enabled the model to accurately predict gene expression in previously unseen cell types. Such integrative frameworks will be critical for capturing the complete cascade from genotype to phenotype.

Another key frontier is causal inference [62]. Most current models identify correlations between sequence patterns and molecular phenotypes, however cannot distinguish cause from association. Embedding causal reasoning—through structural and graphical causal models [63, 64], and experimental perturbations [65, 66]—may enable models to predict the functional impact of variants more reliably and to generate experimentally testable hypotheses about regulatory mechanisms.

Interpretability remains an equally pressing challenge [67]. As genomic models continue to grow in scale and complexity, understanding what they learn becomes critical for their scientific utility. Developing feature attribution, feature interaction, and transparency models that map learned representations back to biological features (e.g. motifs, enhancers, or pathways) will allow these models to serve not merely as predictors but as instruments for biological discovery.

In addition, synthetic data may offer a promising path to overcome the intrinsic limitations of natural genomes. As a recent study [68] has noted, the human genome is relatively small and repetitive, limiting the diversity of regulatory configurations available for model training. MPRAs using synthetic DNA could expand the accessible regulatory landscape, enabling systematic exploration of cis-regulatory grammars. However, how to co-train models on both synthetic and real data remains an open methodological question that will require innovations in domain adaptation and representation alignment.

Finally, sustained progress in this field will hinge on collaboration and resource sharing. Establishing shared benchmarks, promoting cross-institutional data exchange, and pooling computational resources will ensure transparency, reproducibility, and accessibility. Collaborative consortia that unite biologists, computer scientists, and engineers will be vital for translating advances in genomic AI into mechanistic understanding and biomedical impact.

**Key Points**

- Deep learning models achieve high accuracy when predicting molecular phenotypes on reference genomes but fail to robustly predict personalized gene expression across individuals.
- Despite their simpler architecture, linear models are more reliable than deep learning models for cross-individual gene expression prediction.
- Fine-tuned deep learning models show promise for predicting gene expression directly from raw genomic sequences, eliminating the need for predefined biomarkers.
- Genomic language models, which require only sequence-based pretraining, have emerged as a promising alternative for encoding the transcriptional effects of genetic variation.

## Data Availability

No new data were generated or analyzed in support of this research.

## References

[1] Schadt EE, Björkegren JL. New: network-enabled wisdom in biology, medicine, and health care. *Sci Transl Med* 2012; 4:115rv1–115rv1. 10.1126/scitranslmed.3002132 22218693

[2] Albert FW, Kruglyak L. The role of regulatory variation in complex traits and disease. *Nat Rev Genet* 2015; 16:197–212. 10.1038/nrg3891 25707927

[3] Gamazon ER, Wheeler HE, Shah KP et al. A gene-based association method for mapping traits using reference transcriptome data. *Nat Genet* 2015; 47:1091–8. 10.1038/ng.3367 26258848 PMC4552594

[4] Gusev A, Ko A, Shi H et al. Integrative approaches for large-scale transcriptome-wide association studies. *Nat Genet* 2016; 48:245–52. 10.1038/ng.3506 26854917 PMC4767558

[5] Hu Y, Li M, Lu Q et al. A statistical framework for cross-tissue transcriptome-wide association analysis. *Nat Genet* 2019; 51:568–76. 10.1038/s41588-019-0345-7 30804563 PMC6788740

[6] Urbut SM, Wang G, Carbonetto P et al. Flexible statistical methods for estimating and testing effects in genomic studies with multiple conditions. *Nat Genet* 2019;51:187–95. 10.1038/s41588-018-0268-8 30478440 PMC6309609

[7] Zhang Z, Bae YE, Bradley JR et al. Summit: an integrative approach for better transcriptomic data imputation improves causal gene identification. *Nat Commun* 2022; 13:6336. 10.1038/s41467-022-34016-y 36284135 PMC9593997

[8] Barbadilla-Martínez L, Klaassen N, van Steensel B et al. Predicting gene expression from DNA sequence using deep learning models. *Nat Rev Genet* 2025;26:666–80. 10.1038/s41576-025-00841-2 40360798

[9] Chai J, Zeng H, Li A et al. Deep learning in computer vision: a critical review of emerging techniques and application scenarios. *Mach Learn Appl* 2021;6:100134. 10.1016/j.mlwa.2021.100134

[10] Otter DW, Medina JR, Kalita JK. A survey of the usages of deep learning for natural language processing. *IEEE Trans Neural Netw Learn Syst* 2021;32:604–24. 32324570 10.1109/TNNLS.2020.2979670

[11] Sasse A, Ng B, Spiro AE et al. Benchmarking of deep neural networks for predicting personal gene expression from DNA sequence highlights shortcomings. *Nat Genet* 2023;55:2060–4. 10.1038/s41588-023-01524-6 38036778

[12] Huang C, Shuai RW, Baokar P et al. Personal transcriptome variation is poorly explained by current genomic deep learning models. *Nat Genet* 2023;55:2056–9. 10.1038/s41588-023-01574-w 38036790 PMC10703684

[13] Stephan J, Stegle O, Beyer A. A random forest approach to capture genetic effects in the presence of population structure. *Nat Commun* 2015;6:7432. 10.1038/ncomms8432 26109276

[14] Lonsdale J, Thomas J, Salvatore M et al. The genotype-tissue expression (GTEX) project. *Nat Genet* 2013;45:580–5. 23715323 10.1038/ng.2653 PMC4010069

[15] Consortium, EP . An integrated encyclopedia of DNA elements in the human genome. *Nature* 2012;489:57–74. 10.1038/nature11247 22955616 PMC3439153

[16] Kundaje A, Meuleman W, Ernst J et al. Integrative analysis of 111 reference human epigenomes. *Nature* 2015;518:317. 25693563 10.1038/nature14248 PMC4530010

[17] Dekker J, Belmont AS, Guttman M et al. The 4D nucleome project. *Nature* 2017;549:219–26. 10.1038/nature23884 28905911 PMC5617335

[18] Vaswani A, Shazeer N, Parmar N et al. Attention is all you need. *Adv Neural Inform Process Syst* 2017;30:1–15.

[19] Zhou J, Troyanskaya OG. Predicting effects of noncoding variants with deep learning–based sequence model. *Nat Methods* 2015;12:931–4. 10.1038/nmeth.3547 26301843 PMC4768299

[20] Zhou J, Theesfeld CL, Yao K et al. Deep learning sequence-based ab initio prediction of variant effects on expression and disease risk. *Nat Genet* 2018;50:1171–9. 10.1038/s41588-018-0160-6 30013180 PMC6094955

[21] Kelley DR, Reshef YA, Bileschi M et al. Sequential regulatory activity prediction across chromosomes with convolutional neural networks. *Genome Res* 2018;28:739–50. 10.1101/gr.227819.117 29588361 PMC5932613

[22] Agarwal V, Shendure J. Predicting mRNA abundance directly from genomic sequence using deep convolutional neural networks. *Cell Rep* 2020;31:107663. 10.1016/j.celrep.2020.107663 32433972

[23] Avsec Ž, Agarwal V, Visentin D et al. Effective gene expression prediction from sequence by integrating long-range interactions. *Nat Methods* 2021;18:1196–203. 10.1038/s41592-021-01252-x 34608324 PMC8490152

[24] Linder J, Srivastava D, Yuan H et al. Predicting RNA-seq coverage from DNA sequence as a unifying model of gene regulation. *Nat Genet* 2025;57:949–61. 10.1038/s41588-024-02053-6 39779956 PMC11985352

[25] Ronneberger O, Fischer P, Brox T. U-net: convolutional networks for biomedical image segmentation. 2015. http://arxiv.org/abs/1505.04597. ArXiv: 1505.04597.

[26] Avsec Ž, Latysheva N, Cheng J et al. Alphagenome: advancing regulatory variant effect prediction with a unified DNA sequence model. *bioRxiv* 2025;2025–06.

[27] Abugessaisa I, Ramilowski JA, Lizio M et al. Fantom enters 20th year: expansion of transcriptomic atlases and functional annotation of non-coding RNAs. *Nucleic Acids Res* 2021;49:D892–8. 10.1093/nar/gkaa1054 33211864 PMC7779024

[28] Lappalainen T, Sammeth M, Friedländer MR et al. Transcriptome and genome sequencing uncovers functional variation in humans. *Nature* 2013;501:506–11. 10.1038/nature12531 24037378 PMC3918453

[29] Pérez-González AP, García-Kroepfly AL, Pérez-Fuentes KA et al. The Rosmap project: aging and neurodegenerative diseases through omic sciences. *Front Neuroinform* 2024;18:1443865. 10.3389/fninf.2024.1443865 39351424 PMC11439699

[30] Karollus A, Mauermeier T, Gagneur J. Current sequence-based models capture gene expression determinants in promoters but mostly ignore distal enhancers. *Genome Biol* 2023;24:56. 10.1186/s13059-023-02899-9 36973806 PMC10045630

[31] Drusinsky S, Whalen S, Pollard KS. Deep-learning prediction of gene expression from personal genomes. *bioRxiv* 2024;2024–07. 10.1186/s13059-025-03926-7 PMC12869966 41495833

[32] Rastogi R, Reddy AJ, Chung R et al. Fine-tuning sequence-to-expression models on personal genome and transcriptome data. *bioRxiv* 2024;2024–09. 10.1186/s13059-026-04091-1 PMC13386774 42186093

[33] French RM . Catastrophic forgetting in connectionist networks. *Trends Cogn Sci* 1999;3:128–35. 10322466 10.1016/s1364-6613(99)01294-2

[34] Rannon E, Burstein D. Leveraging natural language processing to unravel the mystery of life: a review of NLP approaches in genomics, transcriptomics, and proteomics. 2025. arXiv: 2506.02212.

[35] Zhou Z, Ji Y, Li W et al. DNAbert-2: efficient foundation model and benchmark for multi-species genome. 2023. arXiv: 2306.15006.

[36] Dalla-Torre H, Gonzalez L, Mendoza-Revilla J et al. Nucleotide transformer: building and evaluating robust foundation models for human genomics. *Nat Methods* 2025;22:287–97. 10.1038/s41592-024-02523-z 39609566 PMC11810778

[37] Brixi G, Durrant MG, Ku J et al. Genome modeling and design across all domains of life with evo 2. 2025.02.18.638918. 10.1101/2025.02.18.638918v1 ( Section: New Results). bioRxiv 2025;2025-02.

[38] Ji Y, Zhou Z, Liu H et al. Dnabert: pre-trained bidirectional encoder representations from transformers model for DNA-language in genome. *Bioinformatics* 2021;37:2112–20. 10.1093/bioinformatics/btab083 33538820 PMC11025658

[39] Sanabria M, Hirsch J, Joubert PM et al. DNA language model Grover learns sequence context in the human genome. *Nat Mach Intell* 2024; 6:911–23.

[40] Devlin J, Chang M-W, Lee K et al. Bert: pre-training of deep bidirectional transformers for language understanding. 2019. http://arxiv.org/abs/1810.04805. ArXiv: 1810.04805.

[41] Radford A, Narasimhan K, Salimans T et al. Improving language understanding by generative pre-training. *OpenAI Technical Report* 2018. Pre-print; first GPT model. https://www.mikecaptain.com/resources/pdf/GPT-1.pdf

[42] Radford A, Wu J, Child R et al. Language models are unsupervised multitask learners. *OpenAI Technical Report* 2019. GPT-2, 1.5 B model. https://cdn.openai.com/better-language-models/language_models_are_unsupervised_multitask_learners.pdf

[43] Brown TB, Mann B, Ryder N et al. Language models are few-shot learners. 2020;1877–901. GPT-3, 175 B parameters. arXiv:2005.14165 [cs].

[44] Zhang D, Zhang W, Zhao Y et al. DNAGPT: a generalized pre-trained tool for versatile DNA sequence analysis tasks. 2023 http://arxiv.org/abs/2307.05628. [q-bio]. arXiv:2307.05628.

[45] Schiff Y, Kao C-H, Gokaslan A et al. Caduceus: bi-directional equivariant long-range DNA sequence modeling. 2024. http://arxiv.org/abs/2403.03234. arXiv:2403.03234. PMC12189541 40567809

[46] Hoarfrost A, Aptekmann A, Farfañuk G et al. Deep learning of a bacterial and archaeal universal language of life enables transfer learning and illuminates microbial dark matter. *Nat Commun* 2022;13:2606. 10.1038/s41467-022-30070-8 35545619 PMC9095714

[47] Benegas G, Batra SS, Song YS. DNA language models are powerful predictors of genome-wide variant effects. *Proc Natl Acad Sci* 2023; 120:e2311219120. 10.1073/pnas.2311219120 37883436 PMC10622914

[48] Nguyen E, Poli M, Faizi M et al. HyenaDNA: long-range genomic sequence modeling at single nucleotide resolution. 2023. http://arxiv.org/abs/2306.15794. [cs, q-bio]. arXiv:2306.15794.

[49] Gu A, Gupta A, Goel K et al. On the parameterization and initialization of diagonal state space models. 2022. http://arxiv.org/abs/2206.11893. [cs]. arXiv:2206.11893.

[50] Gu A, Dao T, Ermon S et al. Hippo: Recurrent Memory with Optimal Polynomial Projections. 2020. http://arxiv.org/abs/2008.07669. [cs, stat]. arXiv:2008.07669.

[51] Benegas G, Ye C, Albors C et al. Genomic language models: opportunities and challenges. 2024. http://arxiv.org/abs/2407.11435. arXiv:2407.11435. 10.1016/j.tig.2024.11.013 39753409

[52] Liu T, Zhang X, Ying R et al. Pre-training genomic language model with variants for better modeling functional genomics. *bioRxiv* 2025;2025–02. 10.1038/s44387-026-00103-4 PMC13095650 42022284

[53] Ramprasad P, Pai N, Pan W. Enhancing personalized gene expression prediction from DNA sequences using genomic foundation models. *Hum Genet Genom Adv* 2024;5:100347. 10.1016/j.xhgg.2024.100347 PMC11416237 39205391

[54] Li S, Luo R, Huang Y. Assessing large-scale genomic language models in predicting personal gene expression: promises and limitations. 2025;2025.07.09.664024. 10.1101/2025.07.09.664024v1. bioRxiv 2025;2025-07.

[55] Gu A, Dao T. Mamba: linear-time sequence modeling with selective state spaces. 2023 . http://arxiv.org/abs/2312.00752. arXiv:2312.00752.

[56] Poli M, Wang J, Massaroli S, Quesnelle J. StripedHyena: moving beyond transformers with hybrid signal processing models. 2023. https://github.com/togethercomputer/stripedhyena

[57] Kaplan J, McCandlish S, Henighan T et al. Scaling laws for neural language models. 2020. arXiv:2001.08361.

[58] Poole R, Ohiri E. What Is the Cost of Training Large Language Models? 2025. https://www.cudocompute.com/blog/what-is-the-cost-of-training-large-language-models

[59] Achiam J, Adler S, Agarwal S et al. Gpt-4 Technical Report 2023. http://arxiv.org/abs/2303.08774. arXiv:2303.08774.

[60] xAI . Grok 4. https://x.ai/news/grok-4. 2025 (3 August 2025, date last accessed).

[61] Fu X, Mo S, Buendia A et al. A foundation model of transcription across human cell types. *Nature* 2025;637:965–73. 10.1038/s41586-024-08391-z 39779852 PMC11754112

[62] Hernán MA, Robins JM. Causal Inference: What if, Robins JM, (ed). Boca Raton, FL: Taylor & Francis / Chapman & Hall/CRC, 2024.

[63] Chen LS, Emmert-Streib F, Storey JD. Harnessing naturally randomized transcription to infer regulatory relationships among genes. *Genome Biol* 2007;8:R219. 10.1186/gb-2007-8-10-r219 17931418 PMC2246293

[64] Wang L, Michoel T. Efficient and accurate causal inference with hidden confounders from genome-transcriptome variation data. *PLoS Comput Biol* 2017;13:e1005703. 10.1371/journal.pcbi.1005703 28821014 PMC5576763

[65] Dixit A, Parnas O, Li B et al. Perturb-seq: dissecting molecular circuits with scalable single-cell RNA-profiling of pooled genetic screens. *Cell* 2016;167:1853–1866.e17. 10.1016/j.cell.2016.11.038 27984732 PMC5181115

[66] Rubin AJ, Parker KR, Satpathy AT et al. Coupled single-cell crispr screening and epigenomic profiling reveals causal gene regulatory networks. *Cell* 2019;176:361–376.e17. 10.1016/j.cell.2018.11.022 30580963 PMC6329648

[67] Novakovsky G, Dexter N, Libbrecht MW et al. Obtaining genetics insights from deep learning via explainable artificial intelligence. Nature Reviews Genetics 2023; 24:125–37. https://www.nature.com/articles/s41576-022-00532-2. Number: 2 Publisher: Nature Publishing Group. 10.1038/s41576-022-00532-2 36192604

[68] de Boer CG, Taipale J. Hold out the genome: a roadmap to solving the cis-regulatory code. *Nature* 2024;625:41–50. 10.1038/s41586-023-06661-w 38093018

